# The nuclear phosphoinositide-p53 signalosome in the regulation of cell motility

**DOI:** 10.1093/procel/pwaf043

**Published:** 2025-05-26

**Authors:** Xiaoting Hou, Yu Chen, Bo Zhou, Fengting Liu, Lingyun Dai, Chunbo Chen, Noah D Carrillo, Vincent L Cryns, Richard A Anderson, Jichao Sun, Mo Chen

**Affiliations:** Department of Critical Care Medicine, Shenzhen People’s Hospital (The Second Clinical Medical College, Jinan University; The First Affiliated Hospital, Southern University of Science and Technology), Shenzhen 518020, China; Department of Geriatrics, Guangdong Provincial Clinical Research Center for Geriatrics, Shenzhen Clinical Research Center for Geriatrics, Shenzhen People’s Hospital, Shenzhen 518020, China; Department of Pharmacology, Joint Laboratory of Guangdong–Hong Kong Universities for Vascular Homeostasis and Diseases, SUSTech Homeostatic Medicine Institute, School of Medicine, Southern University of Science and Technology, Shenzhen 518055, China; Department of Critical Care Medicine, Shenzhen People’s Hospital (The Second Clinical Medical College, Jinan University; The First Affiliated Hospital, Southern University of Science and Technology), Shenzhen 518020, China; Department of Geriatrics, Guangdong Provincial Clinical Research Center for Geriatrics, Shenzhen Clinical Research Center for Geriatrics, Shenzhen People’s Hospital, Shenzhen 518020, China; Department of Pharmacology, Joint Laboratory of Guangdong–Hong Kong Universities for Vascular Homeostasis and Diseases, SUSTech Homeostatic Medicine Institute, School of Medicine, Southern University of Science and Technology, Shenzhen 518055, China; Department of Critical Care Medicine, Shenzhen People’s Hospital (The Second Clinical Medical College, Jinan University; The First Affiliated Hospital, Southern University of Science and Technology), Shenzhen 518020, China; Department of Geriatrics, Guangdong Provincial Clinical Research Center for Geriatrics, Shenzhen Clinical Research Center for Geriatrics, Shenzhen People’s Hospital, Shenzhen 518020, China; Department of Pharmacology, Joint Laboratory of Guangdong–Hong Kong Universities for Vascular Homeostasis and Diseases, SUSTech Homeostatic Medicine Institute, School of Medicine, Southern University of Science and Technology, Shenzhen 518055, China; Department of Critical Care Medicine, Shenzhen People’s Hospital (The Second Clinical Medical College, Jinan University; The First Affiliated Hospital, Southern University of Science and Technology), Shenzhen 518020, China; Department of Geriatrics, Guangdong Provincial Clinical Research Center for Geriatrics, Shenzhen Clinical Research Center for Geriatrics, Shenzhen People’s Hospital, Shenzhen 518020, China; Department of Pharmacology, Joint Laboratory of Guangdong–Hong Kong Universities for Vascular Homeostasis and Diseases, SUSTech Homeostatic Medicine Institute, School of Medicine, Southern University of Science and Technology, Shenzhen 518055, China; Department of Critical Care Medicine, Shenzhen People’s Hospital (The Second Clinical Medical College, Jinan University; The First Affiliated Hospital, Southern University of Science and Technology), Shenzhen 518020, China; Department of Geriatrics, Guangdong Provincial Clinical Research Center for Geriatrics, Shenzhen Clinical Research Center for Geriatrics, Shenzhen People’s Hospital, Shenzhen 518020, China; Department of Critical Care Medicine, Shenzhen People’s Hospital (The Second Clinical Medical College, Jinan University; The First Affiliated Hospital, Southern University of Science and Technology), Shenzhen 518020, China; Department of Medicine, School of Medicine and Public Health, University of Wisconsin–Madison, Madison, WI 53705, United States; Department of Medicine, School of Medicine and Public Health, University of Wisconsin–Madison, Madison, WI 53705, United States; University of Wisconsin Carbone Cancer Center, School of Medicine and Public Health, University of Wisconsin–Madison, Madison, WI 53705, United States; University of Wisconsin Carbone Cancer Center, School of Medicine and Public Health, University of Wisconsin–Madison, Madison, WI 53705, United States; Department of Critical Care Medicine, Shenzhen People’s Hospital (The Second Clinical Medical College, Jinan University; The First Affiliated Hospital, Southern University of Science and Technology), Shenzhen 518020, China; Department of Geriatrics, Guangdong Provincial Clinical Research Center for Geriatrics, Shenzhen Clinical Research Center for Geriatrics, Shenzhen People’s Hospital, Shenzhen 518020, China; Department of Pharmacology, Joint Laboratory of Guangdong–Hong Kong Universities for Vascular Homeostasis and Diseases, SUSTech Homeostatic Medicine Institute, School of Medicine, Southern University of Science and Technology, Shenzhen 518055, China; Department of Critical Care Medicine, Shenzhen People’s Hospital (The Second Clinical Medical College, Jinan University; The First Affiliated Hospital, Southern University of Science and Technology), Shenzhen 518020, China; Department of Geriatrics, Guangdong Provincial Clinical Research Center for Geriatrics, Shenzhen Clinical Research Center for Geriatrics, Shenzhen People’s Hospital, Shenzhen 518020, China; Department of Pharmacology, Joint Laboratory of Guangdong–Hong Kong Universities for Vascular Homeostasis and Diseases, SUSTech Homeostatic Medicine Institute, School of Medicine, Southern University of Science and Technology, Shenzhen 518055, China

**Keywords:** phosphoinositide, p53, signalosome, nucleus, cell motility

## Abstract

Dysregulation of p53 and phosphoinositide (PIP_n_) signaling are both key drivers of oncogenesis and metastasis. Our recent findings reveal a previously unrecognized interaction between these pathways, converging in the nucleus to form a PIP_n_-p53 signalosome that modulates nuclear AKT activation and downstream signaling, thereby in”uencing cancer cell survival and motility. This review examines recent insights into nuclear PIP_n_ signaling in the context of established roles for p53 in cell dynamics and migration while also deliberating current research on how nuclear PIP_n_s interact with p53 to form signalosomes that affect cell motility. We emphasize the critical role of PIP_n_s in stabilizing p53 and activating *de novo* nuclear AKT signaling, which subsequently modulates key motility-related pathways. Understanding the unique operation and function of the PIP_n_-p53 signalosome in nuclear phosphatidylinositol 3-kinase (PI3K)-AKT activation offers novel therapeutic strategies for controlling cancer metastasis by targeting pertinent interactions and events.

## Introduction

Cell motility is a fundamental process that, when aberrantly regulated, can lead to the invasive and metastatic characteristics of cancer (Merino-Casallo et al., 2022; Stuelten et al., 2018). The phosphoinositide (PIP_n_) signaling pathways and the tumor suppressor protein p53 are central to the control of this process (Balla, 2013; Hou et al., 2025a; Muller et al., 2011). Independent dysregulation of these pathways is often a key driver in the transition from benign to malignant cell growth and the subsequent spread of cancer (Kandoth et al., 2013; Sinkala, 2023). The p53 protein, revered as the “guardian of the genome,” is a multifaceted regulator of cellular responses to stress, including cell cycle arrest, apoptosis, and DNA repair (Oren and Prives, 2024). Similarly, PIP_n_s, a family of phosphorylated lipids, play pivotal roles in cellular signaling, particularly in the modulation of membrane-associated events and cellular dynamics (Balla, 2013; Thapa et al., 2020, 2024).

Recent discoveries that reveal an intricate interplay between p53 and PIP_n_s within the nucleus of cancer cells have significantly advanced our understanding of these pathways (Chen et al., 2020, 2022; Choi et al., 2019; Ren et al., 2024). This emerging body of work suggests that these two pathways converge to form a nuclear PIP_n_-p53 signalosome. This functional complex regulates nuclear AKT activity and in”uences cancer cell survival and motility (Carrillo et al., 2023; Chen et al., 2022; Choi et al., 2019). The formation of this signalosome represents a novel mechanism through which p53 and PIP_n_s can jointly orchestrate the cellular behavior of cancers, offering overlapping targets for oncogenic phenotypes.

This review presents the latest insights into nuclear PIP_n_ signaling and examines p53’s established roles in regulating cytoskeletal dynamics, cell adhesion, and migration. We integrate recent findings on how nuclear PIP_n_s interact with p53 to form signalosomes that directly in”uence cancer cell motility. Special attention is given to the role of PIP_n_s in stabilizing p53 and activating nuclear AKT signaling, which modulates key pathways essential for cell motility. By highlighting the unique functions of the PIP_n_-p53 signalosome in nuclear phosphatidylinositol 3-kinase (PI3K)-AKT activation, we aim to identify novel therapeutic strategies to control cancer progression and metastasis.

## Recent advances in nuclear PIP_n_ signaling

**Figure 1.**
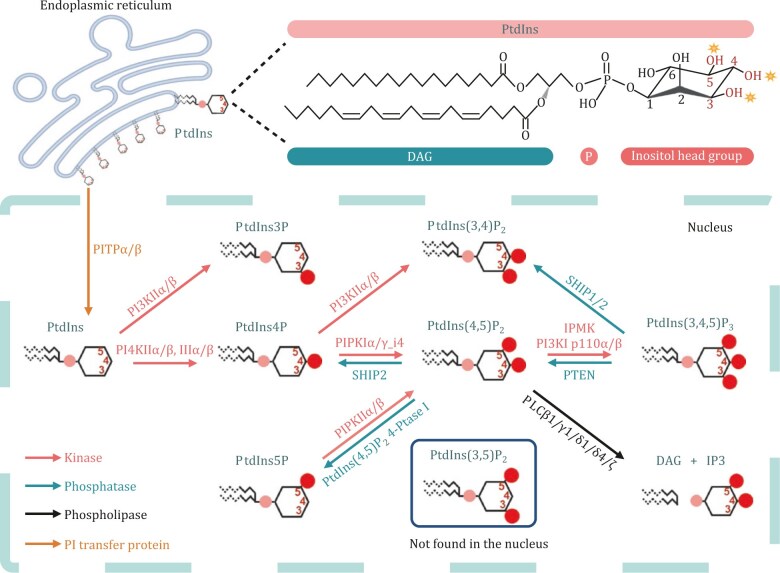
**Nuclear PIP_n_ Metabolism.** Schematic representation of PI/PtdIns structure and the nuclear PIP_n_ cycle. Phosphorylation sites on the hydroxyl groups of the inositol ring are marked by yellow stars, with carbon atom numbers labeled in red. Key components of the nuclear PIP_n_ metabolism, including PI transfer proteins (PITPs), kinases, phosphatases, and phospholipases, are depicted. Abbreviations: IP_3_ (Ins(1,4,5)P_3_), DAG (diacylglycerol), PtdIns(4,5)P_2_ 4-Ptase I (Type I PtdIns(4,5)P_2_ 4-phosphatase). The figure was created using BioRender.

PIP_n_s are phosphorylated derivatives of phosphatidylinositol (PI/PtdIns), a lipid that plays a critical role in cellular signaling. PI comprises a glycerol backbone, two fatty acid acyl chains, and an inositol ring (Fig. 1). The inositol ring can undergo phosphorylation and dephosphorylation at positions 3, 4, and 5 by specific kinases and phosphatases, leading to the generation of seven distinct PIP_n_ isomers. These isomers are PtdIns3P, PtdIns4P, PtdIns5P, PtdIns(3,4)P_2_, PtdIns(3,5)P_2_, PtdIns(4,5)P_2_, and PtdIns(3,4,5)P_3_. These phosphorylated derivatives are central to numerous intracellular signaling pathways regulating cellular functions, such as proliferation, survival, and motility.

PI synthesis occurs in the endoplasmic reticulum (ER) membrane, facilitated by multiple vital enzymes. The process starts with the acylation of glycerol-3-phosphate (G3P) by acyltransferases, producing phosphatidic acid (PA)—the first step in phospholipid biosynthesis across prokaryotes and eukaryotes (Blunsom and Cockcroft, 2020a). PA is then converted into CDP-diacylglycerol (CDP-DAG), a vital liponucleotide intermediate. In eukaryotes, PA serves as a precursor for both CDP-DAG and diacylglycerol (DAG) (Yang et al., 2018). CDP-DAG is crucial for the synthesis of PI, phosphatidylglycerol (PG), and cardiolipin (CL), while DAG is necessary for phosphatidylcholine (PC), phosphatidylethanolamine (PE), and triacylglycerol (TAG) production (Blunsom and Cockcroft, 2020a; Yang et al., 2018). The CDP-diacylglycerol synthase (CDS), also known as CTP:phosphatidate cytidylyltransferase, catalyzes CDP-DAG formation from CTP and PA. In mammals, two CDS enzymes, CDS1 and CDS2, operate in the ER (Blunsom and Cockcroft, 2020b). Following their action, CDP-DAG is utilized by PI synthase (PIS) to generate PI. Both CDS1/2 and PIS are integral ER membrane proteins, confining PI synthesis to the ER (Blunsom and Cockcroft, 2020b).

**Figure 2.**
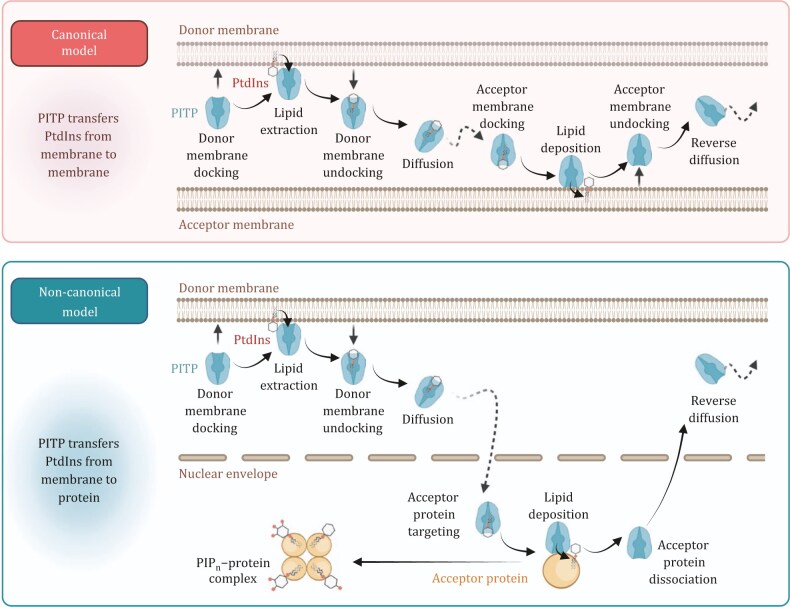
**Canonical and non-canonical models of PITP-mediated lipid transfer.** In the canonical model (top panel), PITP facilitates the transport of PtdIns between membranes. Upon docking at the donor membrane, PITP extracts PtdIns, then undocks and diffuses through the cytosol. It subsequently docks at the acceptor membrane to deliver the lipid. After undocking, PITP diffuses back to repeat the cycle. In the non-canonical model (bottom panel), PITP transfers PtdIns from the membrane to a protein acceptor instead of a membrane. This process follows similar steps—lipid extraction, membrane undocking, and diffusion—but instead of docking onto an acceptor membrane, PITP interacts with a protein acceptor. This interaction results in lipid deposition and the formation of a PIP_n_-protein complex. The cycle completes as PITP dissociates and diffuses back to the donor site. The figure was created using BioRender.

Due to their hydrophobic nature, lipids are generally restricted to cell membranes, preventing their free diffusion within the cell. Specific lipid transfer proteins (LTPs) are required to facilitate their transport (Wong et al., 2019). The subgroup of LTPs responsible for PI transport is known as PI transfer proteins (PITPs). Once PI is synthesized in the ER, PITPs mediate its movement to various cellular compartments (Hsuan and Cockcroft, 2001). In humans, five PITP family members have been identified and classified into two groups: class I, which includes PITPα and PITPβ, and class II, which comprises phosphatidylinositol transfer protein cytoplasmic 1 (PITPNC1), membrane-associated phosphatidylinositol transfer protein 1 (PITPNM1), and PITPNM2 (Hsuan and Cockcroft, 2001). Historically, PIs were thought to be restricted to the plasma membrane and endomembrane compartments for cytoplasmic signaling (Posor et al., 2022). According to the canonical model, PITPs shuttle PI between membranes in a countercurrent manner, often transporting an additional lipid cargo (Wong et al., 2019). Class I PITPs exchange PI and PC between membranes, while class II PITPs transfer PI and PA between membrane structures (Hsuan and Cockcroft, 2001) (Fig. 2).

Since the initial discovery of nuclear PIs in 1965, it has become evident that PIs are present in the nucleus and play crucial roles in regulating key cellular processes (Chen et al., 2020; Rose and Frenster, 1965; Tribble et al., 2016). Our recent findings reveal that class I PITPs, PITPα and PITPβ, localize to the nucleus in response to cellular stress, where they play a dominant role in establishing the nuclear PIP_n_ pool (Carrillo et al., 2023; Wen et al., 2024). This suggests lipid transfer proteins translocate PI from the ER to the nucleus. PITP executes lipid vectorial transport by establishing a stereochemically constrained microenvironment: its hydrophobic substrate-binding domain encapsulates fatty acyl chains while precisely positioning the inositol moiety at the solvent interface (Wong et al., 2019). This dual spatial organization enables concurrent fulfillment of metabolic imperatives, stabilizing the labile phospholipid during transmembrane transit while presenting an orientation-locked phosphorylation platform for nuclear kinase recognition. Prior to these studies, PITPs were rarely considered in the context of the nucleus, particularly concerning non-membrane regions within the nucleoplasm. In the canonical model, lipid transfer proteins, including PITPs, are proposed to transfer lipids from membrane to membrane (Fig. 2). The discovery that PITPs mediate lipid transfer from membrane- bound structures to the nucleoplasm challenges the traditional view of lipid transfer, which was thought to be confined to membrane structures. In the updated non-canonical model, PITPs could transfer lipids from the membrane to non-membrane structures, including their protein targets. Consistent with the nuclear presence of PITPs, various PIP_n_ species—including PtdIns3P, PtdIns4P, PtdIns5P, PtdIns(3,4)P_2_, PtdIns(4,5)P_2_, and PtdIns(3,4,5)P_3_—have been identified in the nucleus, with PtdIns(3,5)P_2_ being the only exception (Chen et al., 2020). This expands the understanding of lipid signaling within nuclear domains, underscoring its significance in nuclear functions and cellular stress responses.

In 1983, PI kinase and PIP_n_ kinase activity were first detected in the nucleus, supporting the presence of PI-modifying enzymes that convert specific PIP_n_s into their phosphorylated forms (Barlow et al., 2010; Boronenkov et al., 1998; Chen et al., 2020; Cocco et al., 1987; Manzoli et al., 1977; Rose and Frenster, 1965; Smith and Wells, 1983). Subsequent studies have established the nuclear localization and activity of PI/PIP_n_ kinases, phosphatases, phospholipases, and downstream PIP_n_ effectors, indicating the existence of a dynamic pool of nuclear PIP_n_s independent of cytoplasmic stores (Chen et al., 2020; Cocco et al., 1987; Faenza et al., 2013). These nuclear PIP_n_s are synthesized from nuclear PI and metabolized in the nucleus, pointing to a membrane-independent PIP_n_ signaling network in the nucleoplasm. Nuclear PIP_n_s play essential roles in DNA repair, chromatin remodeling, and gene expression, and they are critical for genome stability and cell fate determination (Barlow et al., 2010; Gozani et al., 2003; Sztacho et al., 2019). As shown in Table 1, the nuclear-localized PITPs and PI-metabolizing enzymes, including PIP_n_ kinases, phosphatases, and phospholipases, are all integral to regulating cell motility. Their nuclear localization is likely a critical factor in their ability to modulate cell motility, underscoring the importance of further investigation into this regulatory mechanism.

**Table 1.** The role of nuclear PI transfer proteins and metabolizing enzymes in cell motility.

**Nuclear PI transfer proteins**				
**Gene name**	**Courier**	**Cargo**	**Nuclear location**	**Role in cell motility**
PITPNA	PITPα	PtdIns (Ashlin et al., 2021), PC (Ashlin et al., 2021)	Nucleoplasm (Carrillo et al., 2023; Wen et al., 2024), nuclear speckles (Carrillo et al., 2023; Wen et al., 2024)	Collaborate with PITPβ to establish the nuclear PI pool, promoting cancer cell proliferation, migration, and invasion (Carrillo et al., 2023)
PITPNB	PITPβ	PtdIns (Ashlin et al., 2021), PC (Ashlin et al., 2021)	Nucleoplasm (Carrillo et al., 2023; Wen et al., 2024), nuclear speckles (Carrillo et al., 2023; Wen et al., 2024)	Collaborate with PITPα to establish the nuclear PI pool, promoting cancer cell proliferation, migration, and invasion (Carrillo et al., 2023)
**Nuclear PIP_n_ kinases**				
**Gene name**	**Enzyme**	**Substrate**	**Nuclear location**	**Role in cell motility**
PI4K2A	PI4KIIα	PtdIns (Balla and Balla, 2006)	Nucleus (Kakuk et al., 2006), nucleoplasmic Ca^2+^ store vesicles (Yoo et al., 2014), nucleoplasm (Carrillo et al., 2023; Wen et al., 2024), nuclear speckles (Carrillo et al., 2023; Chen et al., 2022; Wen et al., 2024)	Mediate cancer cell proliferation, migration, and invasion (Carrillo et al., 2023; Chen et al., 2022; Isaji et al., 2019; Li et al., 2017; Wen et al., 2024)
PI4K2B	PI4KIIβ	PtdIns (Balla and Balla, 2006)	Nucleoplasmic Ca^2+^ store vesicles (Yoo et al., 2014)	Facilitate cancer cell invasion (Alli-Balogun et al., 2016)
PI4KA	PI4KIIIα	PtdIns (Balla and Balla, 2006)	Nucleoplasm (Kakuk et al., 2006), nucleoli (Kakuk et al., 2006, 2008)	Facilitate cancer cell proliferation, migration, and invasion (Govindarajan et al., 2023; Sbrissa et al., 2019; Tran et al., 2024)
PI4KB	PI4KIIIβ	PtdIns (Balla and Balla, 2006)	Nuclear lamina-pore complexes (de Graaf et al., 2002), nuclear speckles (Szivak et al., 2006)	Facilitate cancer cell proliferation (Morrow et al., 2014; Tan et al., 2020)
PIP5K1A	PIPKIα	PtdIns4P (Choi et al., 2019)	Nuclear matrix (Barlow et al., 2010), nucleoplasm (Chen et al., 2022; Choi et al., 2019), nuclear speckles (Carrillo et al., 2023; Chen et al., 2022; Wen et al., 2024), nucleoli (Chakrabarti et al., 2015), chromatin (Choi et al., 2019)	Facilitate cancer cell proliferation, migration, and invasion (Carrillo et al., 2023; Chen et al., 2022; Choi et al., 2019; Wen et al., 2024)
PIP5K1C	PIPKIγ_i4	PtdIns4P (Schill and Anderson, 2009)	Nuclear matrix (Barlow et al., 2010), nuclear speckles (Schill and Anderson, 2009)	Facilitate cancer cell migration, proliferation, and invasion (Sun et al., 2010)
PIP4K2A	PIPKIIα	PtdIns5P (Boronenkov et al., 1998)	Nuclear matrix (Barlow et al., 2010), nuclear speckles (Boronenkov et al., 1998)	Facilitate cancer cell proliferation (Choi et al., 2019)
PIP4K2B	PIPKIIβ	PtdIns5P (Kouchi et al., 2011)	Nuclear matrix (Barlow et al., 2010), nuclear speckles (Boronenkov et al., 1998; Bunce et al., 2008), chromatin (Kouchi et al., 2011; Stijf-Bultsma et al., 2015)	Inhibit cancer cell migration and invasion (Kouchi et al., 2011)
**Nuclear PIP_n_ kinases**				
PIK3R1	Class I PI3K, p85α	-	Nucleus (Tanaka et al., 1999)	Complex with p110α to regulate cancer cell proliferation (Thapa et al., 2020, 2024; Vallejo-Díaz et al., 2019)
PIK3R2	Class I PI3K, p85β	-	Nucleus (Kumar et al., 2011)	Complex with p110α to regulate cancer cell proliferation (Hao et al., 2022; Thapa et al., 20202024)
PIK3CA	Class I PI3K, p110α	PtdIns(4,5)P_2_ (Jean and Kiger, 2014)	Nucleus (Marques et al., 2009), chromatin (Marques et al., 2009)	Complex with p85α/p85β to control cancer cell proliferation (Thapa et al., 2020, 2024)
PIK3CB	Class I PI3K, p110β	PtdIns(4,5)P_2_ (Jean and Kiger, 2014)	Nucleoli (Karlsson et al., 2016), chromatin (Marques et al., 2009)	Facilitate cancer cell proliferation (Mazloumi Gavgani et al., 2018)
PIK3C2A	Class IIα PI3K	PtdIns (Jean and Kiger, 2014), PtdIns4P (Jean and Kiger, 2014)	Nuclear speckles (Didichenko and Thelen, 2001)	Facilitate cancer cell proliferation and migration (Elis et al., 2008; Seok et al., 2010)
PIK3C2B	Class IIβ PI3K	PtdIns (Jean and Kiger, 2014), PtdIns4P (Jean and Kiger, 2014)	Nuclear envelope (Visnjic et al., 2002), nuclear matrix (Banfic et al., 2009; Sindić et al., 2001)	Facilitate cancer cell proliferation (Russo et al., 2015)
IPMK	IPMK	PtdIns(4,5)P_2_ (Blind et al., 2012)	Chromatin (Blind et al., 2012; Xu et al., 2013), nucleoplasm (Carrillo et al., 2023; Chen et al., 2022; Wen et al., 2024), nuclear speckles (Carrillo et al., 2023; Chen et al., 2022; Wen et al., 2024)	Contribute to cancer cell proliferation, migration, and invasion (Carrillo et al., 2023; Chen et al., 2022; Wen et al., 2024)
**Nuclear PIP_n_ phosphatases**				
**Gene name**	**Enzyme**	**Substrate**	**Nuclear location**	**Role in cell motility**
PTEN	PTEN	PtdIns(3,4,5)P_3_ (Deleris et al., 2003)	Nucleoli (Li et al., 2014), chromatin (Choi et al., 2013; Shen et al., 2007), nucleoplasm (Carrillo et al., 2023; Chen et al., 2022; Wen et al., 2024), nuclear speckle (Carrillo et al., 2023; Chen et al., 2022; Wen et al., 2024)	Inhibit cancer cell proliferation, migration, and invasion (Carrillo et al., 2023; Chen et al., 2022; Wen et al., 2024)
INPP5D	SHIP1	PtdIns(3,4,5)P_3_ (Ehm et al., 2015)	Nucleoli (Ehm et al., 2015)	Inhibit cancer cell proliferation (Pedicone et al., 2021)
INPPL1	SHIP2	PtdIns(3,4,5)P_3_ (Deleris et al., 2003), PtdIns(4,5)P_2_ (Elong Edimo et al., 2011)	Nuclear speckles (Deleris et al., 2003; Elong Edimo et al., 2011)	Inhibit cancer cell proliferation (Pedicone et al., 2021)
PIP4P1	Type I PtdIns(4,5)P_2_ 4-phosphatase	PtdIns(4,5)P_2_(Zou et al., 2007)	Nucleus (Zou et al., 2007)	Unknown
**Nuclear phospholipases**				
**Gene name**	**Enzyme**	**Substrate**	**Nuclear location**	**Role in cell motility**
PLCB1	PLCβ1	PtdIns(4,5)P_2_ (Neri et al., 2002)	Nuclear matrix (Cocco et al., 1999), nuclear speckles (Tabellini et al., 2003)	Facilitate cancer cell migration (Owusu Obeng et al., 2020)
PLCG1	PLCγ1	PtdIns(4,5)P_2_ (Lattanzio et al., 2019)	Nucleus (Lattanzio et al., 2019)	Facilitate cancer cell proliferation (Lattanzio et al., 2019; Owusu Obeng et al., 2020; Razmara et al., 2013)
PLCD1	PLCδ1	PtdIns(4,5)P_2_ (Stallings et al., 2005)	Nuclear matrix (Stallings et al., 2005)	Inhibit cancer cell migration and invasion (Owusu Obeng et al., 2020)
PLCD4	PLCδ4	PtdIns(4,5)P_2_ (Kunrath-Lima et al., 2018)	Nucleus (Kunrath-Lima et al., 2018)	Facilitate cancer cell proliferation and migration (Owusu Obeng et al., 2020)
PLCZ1	PLCζi	PtdIns(4,5)P_2_ (Kunrath-Lima et al., 2018)	Nucleus (Faenza et al., 2013; Sone et al., 2005)	Facilitate cancer cell proliferation and migration (Li and Luan, 2018)

This table provides an overview of the types of couriers/enzymes, their respective cargo/substrates, and specific nuclear locations for PITPs and PI-metabolizing enzymes, including PIP_n_ kinases, phosphatases, and phospholipases. It also details the roles of these proteins in cell motility. Their nuclear localization likely contributes to their role in regulating cell motility, a function that warrants further investigation.

## The role of p53 in cell dynamics

The tumor suppressor protein p53, encoded by the *TP53* gene, is the most well-known protein for maintaining cellular integrity (Agarwal et al., 1998; Hollstein et al., 1991; May and May, 1999). It regulates a wide range of cellular processes, including cell cycle arrest, apoptosis, senescence, DNA repair, and metabolism (Agarwal et al., 1998; Hassin and Oren, 2023). By orchestrating these functions, p53 serves as a critical defense mechanism against oncogenesis, preventing the proliferation of cells with damaged DNA (Hassin and Oren, 2023; Hollstein et al., 1991; May and May, 1999).

Under normal physiological conditions, p53 levels are kept low through interaction with mouse double minute 2/4 (MDM2/4), an E3 ubiquitin ligase that ubiquitinates p53, leading to its degradation via the proteasome (Hassin and Oren, 2023; Kruse and Gu, 2009; Kubbutat et al., 1997). This interaction serves as a critical regulatory mechanism preventing inappropriate activation of p53 under basal conditions. However, in response to various stress signals, such as DNA damage, oxidative stress, and oncogene activation, p53 undergoes a series of post-translational modifications that result in its stabilization and activation (Hollstein et al., 1991; Kruse and Gu, 2009; Kubbutat et al., 1997; Mantovani et al., 2019). These modifications include phosphorylation, acetylation, and SUMOylation, which disrupt the p53-MDM2 interaction and enhance p53’s transcriptional activity. Upon activation, p53 operates as a transcription factor by binding to specific DNA response elements, thereby inducing the expression of target genes encoding p21, Ku86, miR-34a, Fas, Bax, and others (Li et al., 2002; Meza-Sosa et al., 2022; Okazaki, 2022; Sigalotti et al., 2010). These genes are crucial for cell cycle arrest, DNA repair, and apoptosis, thus positioning p53 as a central regulator in maintaining genomic integrity and preventing tumorigenesis.

**Figure 3.**
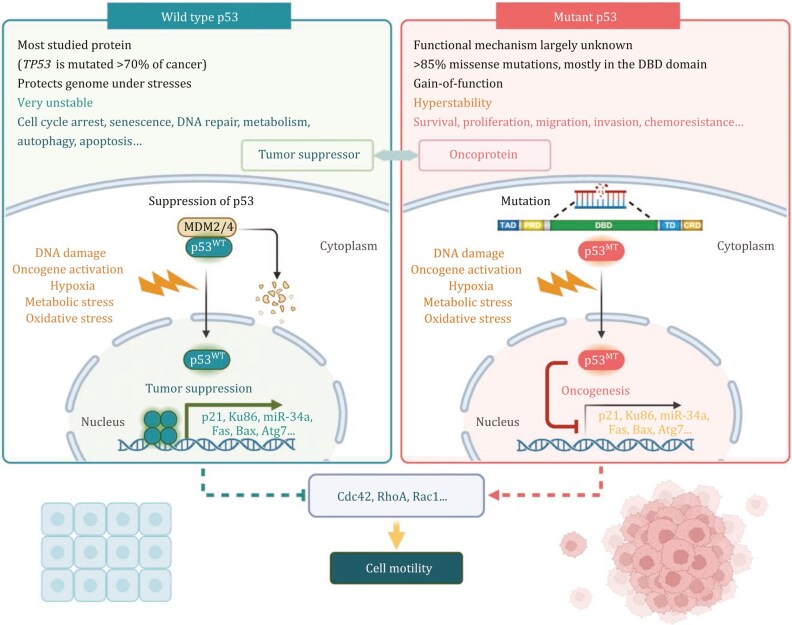
**Functional comparison of wild type and mutant p53.** Schematic representation compares wild type p53 (p53^WT^) and mutant p53 (p53^MT^) roles. Wild type p53, encoded by the *TP53* gene, is one of the most extensively studied proteins and is mutated in over 70% of cancers. Under normal conditions, p53^WT^ is highly unstable, as it binds to MDM2/4, leading to its ubiquitination and degradation. Upon cellular stress, p53^WT^ translocates to the nucleus, forms a tetramer, and functions as a transcription factor. It activates the expression of genes encoding p21, Ku86, miR-34a, Fas, Bax, and Atg7, which promote cell cycle arrest, senescence, DNA repair, metabolism, autophagy, and apoptosis. In contrast, p53^MT^ loses its transcriptional activity but exhibits gain-of-function properties. With over 85% of mutations being missense, mainly in the DNA-binding domain (DBD), p53^MT^ becomes hyperstable. It is constitutively expressed at basal levels and further upregulated under stress, functioning as an oncoprotein that promotes cancer cell survival, proliferation, migration, invasion, and chemoresistance. The proposed roles of both wild type and mutant p53 in tumor suppression and oncogenesis could affect critical regulators of cell dynamics, such as Cdc42, RhoA, and Rac1, thereby modulating cell motility. The figure was created using BioRender.

Mutations in the *TP53* gene are the most common genetic alterations found in human cancers, occurring in nearly every type of cancer, such as lung, breast, colon, and ovarian cancers (Hollstein et al., 1991; Langerød et al., 2007; Olivier et al., 2010; Wang et al., 2004a, 2004b). These mutations can lead to various outcomes, primarily resulting in a loss of p53’s transcriptional activity and tumor-suppressive functions (Hollstein et al., 1991; Olivier et al., 2010). In many cases, mutated p53 proteins acquire dominant-negative properties, where they fail to transcribe target genes and inhibit the activity of any remaining wild-type p53 (de Vries et al., 2002; Kennedy and Lowe, 2022). This interference allows cancer cells to bypass critical regulatory checkpoints, evading growth control mechanisms and apoptotic pathways that would generally curtail their proliferation (Butera and Amelio, 2024; de Vries et al., 2002; Kennedy and Lowe, 2022). The pervasive nature of *TP53* mutations underscores the importance of p53 as a guardian of the genome and highlights its central role in cancer biology (Fig. 3).

Beyond its role in regulating cell survival, p53 also prominently contributes to cancer cell motility, which is fundamental to the mechanisms of cancer metastasis (Walerych et al., 2012, 2015). Cell motility, the ability of cells to move, is tightly regulated in healthy cells; however, in cancer, this process becomes dysregulated, enabling cancerous cells to invade surrounding tissues and metastasize to distant sites (Stuelten et al., 2018). p53 affects cell motility by in”uencing several vital mechanisms, including cytoskeleton regulation, epithelial- to-mesenchymal transition (EMT), and control of cell adhesion (Araki et al., 2015; Chang et al., 2011; Coutts et al., 2009; Muller et al., 2011; Yeudall et al., 2013). Through regulation of actin filament dynamics, p53 helps maintain cytoskeletal stability, preventing overactive rearrangements associated with increased cell motility. Specifically, p53 modulates cell motility by impacting the activity of key Rho GTPases, including RhoA, Rac1, and Cdc42, which are essential regulators of actin cytoskeletal dynamics (Araki et al., 2015; Gadea et al., 2007; Gadéa et al., 2002; Guo et al., 2003; Mizuarai et al., 2006; Muller et al., 2011). These GTPases control various aspects of actin polymerization and organization, contributing to forming cellular protrusions such as lamellipodia and filopodia, which are critical for cell movement (Charest and Firtel, 2007; Gadéa et al., 2002; Srinivasan et al., 2003). By inhibiting the activity of RhoA, wild-type p53 reduces stress fiber formation, promoting a more rounded cellular morphology that is less conducive to invasive behavior (Gadea et al., 2007).

Conversely, mutant p53 can indirectly activate RhoA through the intermediary of guanine nucleotide exchange factor-H1 (GEF-H1), which enhances the directional migration of cells (Mizuarai et al., 2006). Furthermore, the wild-type p53 protein exerts an inhibitory effect on the activity of Cdc42, consequently impeding the formation of filopodia and suppressing cell motility (Gadéa et al., 2002). Many mutant p53 variants engage in a molecular interaction with Rac1, consequently impeding the association between Rac1 and the SENP1 (SUMO-specific protease 1). This interaction abrogates the SENP1-dependent de-SUMOylation of Rac1, a prerequisite for activating Rac1, thereby facilitating tumor progression (Yue et al., 2017). Moreover, p53’s regulation of these GTPases is crucial for maintaining the balance between motility and adhesion, ensuring that cells do not become overly mobile, which could lead to local and distant metastasis. This dual role highlights p53’s importance in tumor suppression and in regulating the delicate interplay between cell adhesion and motility, further demonstrating diverse therapeutic avenues to explore in the treatment of cancers with mutant p53.

**Figure 4.**
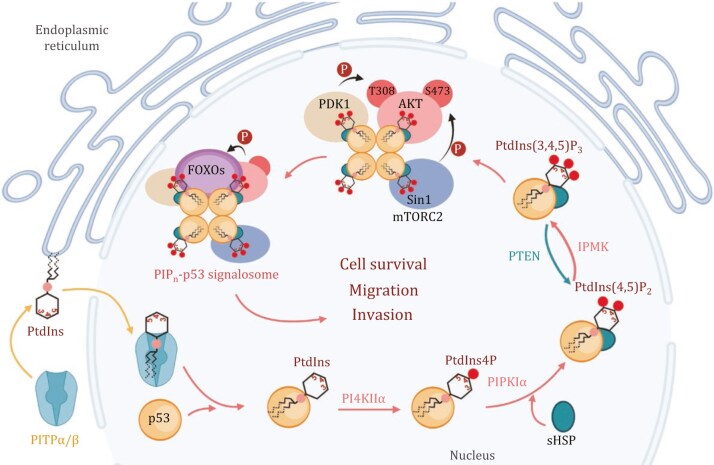
**Nuclear PIP_n_-p53 signalosome assembly and function in cell survival and motility.** The nuclear PIP_n_-p53 signalosome, a key regulator of cancer cell survival and motility, is assembled through interactions between PIP_n_s, PITPs, kinases, phosphatases, p53, and downstream nuclear effectors. Critical steps in its formation include PI transport to the nucleus by class I PITPs, phosphorylation to PtdIns4P by PI4KIIα, and the generation of PtdIns(4,5)P_2_ and PtdIns(3,4,5)P_3_ by PIPKIα and IPMK, respectively. These lipids stabilize p53 and activate nuclear AKT, affecting cell survival and motility. Regulation by wild-type p53 ensures cellular homeostasis, while mutant p53 drives constitutive AKT activation and oncogenic processes. PTEN counteracts this by dephosphorylating PtdIns(3,4,5#P_3_ to PtdIns(4,5)P_2_, preventing further AKT activation. This model highlights the pivotal role of the nuclear PIP_n_-p53 signalosome in modulating cancer cell behavior. The figure was created using BioRender.

In summary, p53 is critical in controlling cancer cell behavior, particularly in preventing cell motility and metastasis. However, when p53 is mutated or dysregulated, these protective mechanisms are lost, leading to increased cancer progression and metastasis and connecting p53 dysfunction to many hallmarks of aggressive cancers. Understanding the role of p53 in regulating cancer cell motility is vital for developing targeted therapies to restore its wild-type function and mitigate mutant oncogenic functions to prevent metastatic disease.

## Assembly of the nuclear PIP_n_-p53 signalosome

Within the nucleus, PIP_n_s interact with various nuclear proteins, including p53, speckle-targeted PIPKIα-regulated poly(A) polymerase (Star-PAP), steroidogenic factor-1 (SF-1), nuclear factor erythroid 2-related factor 2 (NRF2), and Hippo pathway effectors such as yes-associated protein 1 (YAP), to form functional signalosomes that regulate essential cellular processes (Chen et al., 2024; Enomoto et al., 2005; Jung et al., 2024; Mellman et al., 2008; Wang and Sheetz, 2022). The nuclear PIP_n_-p53 signalosome, in particular, plays a critical role in modulating cancer cell motility and invasion.

Our recent findings revealed that the PIP_n_-p53 signalosome is central to regulating cancer cell motility, especially during cancer progression and metastasis, when cell movement and invasion into surrounding tissues are crucial (Chen et al., 2021, 2022; Choi et al., 2019). This signalosome is formed by interacting with various PIP_n_ species, kinases, phosphatases, and lipid effectors, with both wild-type and mutant p53 in the nucleus (Ren et al., 2024) (Fig. 4).

The assembly of the nuclear PIP_n_-p53 signalosome begins with the transport of PI from the ER to the nucleus by PITPs. Notably, class I PITP” was identified as a significant component of the p53 interactome (Huang et al., 2012), and we later demonstrated that both class I PITPα and PITPβ interact with p53 inside the nucleus in a stress-responsive manner (Carrillo et al., 2023). The stress-induced nuclear accumulation of PIP_n_s is primarily driven by class I PITPs, while class II PITPs, including PITPNC1, PITPNM1, and PITPNM2, play a minimal role in maintaining or inducing nuclear PIP_n_ pools (Carrillo et al., 2023; Wen et al., 2024).

Once in the nucleus, PI complexed with p53 recruits PI kinase PI4KIIα to initiate signaling by phosphorylating PI into PtdIns4P (Carrillo et al., 2023). This newly generated PtdIns4P further recruits phosphatidylinositol phosphokinase type I alpha (PIPKIα) under conditions of cellular stress, which phosphorylates PtdIns4P to produce PtdIns(4,5)P_2_ directly linked to p53 (Choi et al., 2019). PtdIns(4,5)P_2_ generation stabilizes p53 by facilitating its interaction with molecular chaperones HSP27 (HSPB1) and αB-crystallin (HSPB5) (Choi et al., 2019). These interactions are crucial for maintaining the nuclear stability of p53 under stress as inhibition of PIPKIα activity or disruption of PtdIns(4,5)P_2_ binding to p53 results in the destabilization of nuclear p53, highlighting the importance of this pathway in maintaining p53 functionality.

The triphosphate form of PI, PtdIns(3,4,5)P_3_, generated at the signalosome complex on p53 in the nucleus, activates nuclear AKT in response to genotoxic stress via a unique p53-dependent mechanism (Chen et al., 2022). When exposed to genotoxic stress, nuclear inositol polyphosphate multikinase (IPMK) associates with p53 in non-membrane nucleoplasm, forming a complex that includes p53 and PtdIns(3,4,5)P_3_ (Chen et al., 2022). This complex recruits key signaling molecules dependent on PtdIns(3,4,5)P_3_ binding, such as phosphoinositide- dependent kinase 1 (PDK1), which phosphorylates AKT at threonine 308, and mammalian target of rapamycin complex 2 (mTORC2), which phosphorylates AKT at serine 473. This process fully activates AKT in the nucleus. Once activated, AKT phosphorylates forkhead box O (FOXO) proteins, leading to FOXO degradation and the subsequent suppression of DNA damage-induced apoptosis (Chen et al., 2022).

The activation of nuclear AKT is tightly regulated by wild-type p53, which modulates AKT activation in response to stress stimuli (Chen et al., 2022). In contrast, mutant p53 results in consistently elevated basal AKT activity, which is dose-dependent and contributes to oncogenic processes (Carrillo et al., 2023; Chen et al., 2022). The PtdIns(3,4,5)P_3_-p53 complex is eventually dephosphorylated by phosphatase and tensin homolog deleted on chromosome ten (PTEN), converting it into a PtdIns(4,5)P_2_-p53 complex that is insufficient for PDK1 and mTORC2 recruitment preventing further AKT activation (Chen et al., 2022).

## Regulation of the nuclear PIP_n_-p53 signalosome on cell motility

PI is transported into the nucleus by class I PITPs, contributing to forming a nuclear PIP_n_ pool (Carrillo et al., 2023; Wen et al., 2024). Within the nucleus, its downstream metabolites—PtdIns4P, PtdIns(4,5)P_2_, and PtdIns(3,4,5) P_3_—along with the enzymes responsible for their synthesis, are also present (Chen et al., 2021, 2022; Choi et al., 2019). In this context, p53 functions as a nuclear scaffolding protein, analogous to the cytosolic scaffold protein IQ motif-containing GTPase-activating protein 1 (IQGAP1) platform (Chen et al., 2019; Choi et al., 2016), assembling a signalosome with PIP_n_s. This PIP_n_-p53 signalosome regulates p53 stability and activates the AKT signaling pathway within the nucleus, thereby modulating various cellular processes (Fig. 4).

AKT signaling is a crucial pathway in various cellular processes, including cell growth, survival, proliferation, and metabolism (Cingolani and Goda, 2008; Ke et al., 2024; Vara et al., 2004). Human AKT comprises three iso-forms (AKT1–3), each potentially serving distinct functions (Gonzalez and McGraw, 2009). Traditionally, AKT (also known as protein kinase B) has been studied in its roles at the plasma membrane, where it is activated by PIP_n_ signaling, particularly by PtdIns(3,4,5)P’ generated by PI3K. However, further works have identified the existence of intranuclear AKT, which extends the functional repertoire of AKT beyond its classical cytoplasmic roles (Lee et al., 2008; Wainstein et al., 2022).

Since the 1990s, accumulating evidence has demonstrated that AKT is localized within the nucleus, with all three isoforms exhibiting a classic leucine-rich, leptomycin- sensitive nuclear export sequence (NES) (Ahmed et al., 1993; Meier et al., 1997; Saji et al., 2005). Notably, AKT is highly expressed in thyroid cancer, and its expression and localization correlate closely with cancer cell invasion and migration in this context (Vasko et al., 2004). Research by Ehud Wainstein et al. indicated that in breast cancer cells, AKT3 is constitutively phosphorylated at the nuclear membrane, facilitating the continuous phosphorylation of tuberous sclerosis complex 2 (TSC2) at this site (Wainstein et al., 2022). Moreover, the knockdown of AKT3 resulted in a moderate reduction in breast cancer cell proliferation. In non-small cell lung cancer (NSCLC), ionizing radiation (IR)-induced activation of nuclear AKT has been shown to depend significantly on human epidermal growth factor receptor 3 (HER3) expression (Toulany et al., 2022).

Furthermore, in PC12 cells, nuclear AKT interacts with nucleophosmin (NPM/B23), a protein that regulates cell growth and apoptosis, modulating its stability and activity (Lee et al., 2008). This interaction protects B23 from degradation, promotes cell survival, and in”uences cell cycle progression, with AKT2 specifically governing B23 SUMOylation. These findings underscore the multifaceted roles of intranuclear AKT in cancer biology, emphasizing its critical importance in regulating processes, such as cell survival, proliferation, and migration, which are essential for cancer progression and metastasis.

Nuclear AKT is instrumental in modulating cancer cell motility by in”uencing the dynamics of the actin cytoskeleton, which is crucial for cell migration (Cheng et al., 2008; Manning and Toker, 2017; Martelli et al., 2012; Sale and Sale, 2008). Studies have demonstrated that AKT in”uences cell proliferation, division, and invasion by modulating downstream effectors such as mTORC2 and Rho GTPases (Enomoto et al., 2005; Xue and Hemmings, 2013; Yoeli-Lerner et al., 2005). While AKT has been identified as a nuclear protein with significant functional implications, the mechanisms underlying its activation within the nucleus have been controversial. Our research offers a novel perspective on the nuclear activation of AKT, emphasizing its relevance in this framework. The PIP_n_-p53 signalosome activates the AKT signaling pathway independent of clinically targeted PI3Ks, impacting cellular activities through downstream signaling cascades.

**Figure 5.**
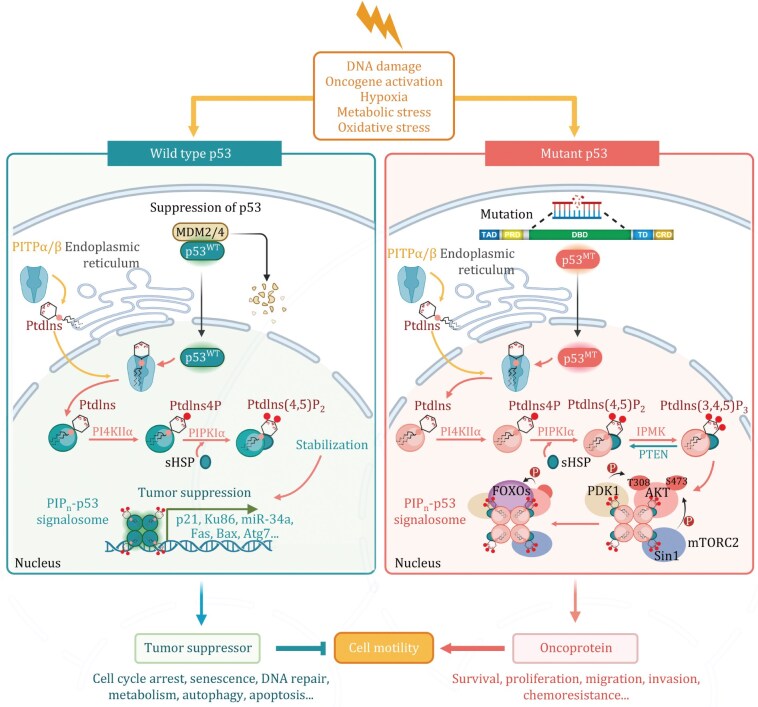
**The nuclear PIP_n_-p53 signalosome in regulating cell motility.** This schematic illustrates the pivotal role of the nuclear PIP_n_-p53 signalosome in regulating both p53^WT^ and p53^MT^ functions, with direct implications for cell motility. Under normal conditions, p53^WT^ is unstable due to its interaction with MDM2/4, leading to its ubiquitination and degradation. In response to stress, class I PITPα/β shuttles PI to the nucleus with p53^WT^, forming the PIP_n_-p53 signalosome. The binding of PtdIns(4,5)P_2_ recruits sHSPs, stabilizing p53 and facilitating tetramer assembly. As a transcription factor, p53^WT^ activates genes that regulate cell cycle arrest, senescence, DNA repair, metabolism, autophagy, and apoptosis. In contrast, p53^MT^ forms a constitutive complex with PIP_n_s, creating the PIP_n_-p53 signalosome. Upon stress, nuclear targeting of class I PITPα/β enhances this signalosome formation, further stabilizing p53^MT^. This also induces the generation of the PtdIns(3,4,5)P_3_ pathway linked to p53^MT^, leading to *de novo* activation of the nuclear AKT pathway, which drives oncogenesis, such as cancer cell survival, proliferation, migration, invasion, and chemoresistance. This graphical abstract underscores the central role of the nuclear PIP_n_-p53 signalosome in modulating p53-mediated cellular functions, including cell motility. The figure was created using BioRender.

Moreover, the stabilization of p53 within the nucleus, facilitated by PtdIns(4,5)P_2_ and its associated small heat shock proteins, inhibits uncontrolled cell migration and invasion (Choi et al., 2019). When p53 is functional, it helps suppress EMT, a process by which cancer cells lose their epithelial characteristics and gain migratory properties (Chang et al., 2011; Coutts et al., 2009; Yeudall et al., 2013). However, when this signalosome is disrupted, either by mutations in p53 or alterations in nuclear PIP_n_ signaling, it can enhance cell motility and contribute to the invasive potential of metastatic cancer.

In summary, intranuclear PIP_n_s play an essential role in maintaining the stability of p53 under stress. In this process, p53 acts as a scaffolding protein whereby PI-related enzymes and effectors form a complex with p53. PIP_n_s affect the stability of p53 by creating an intricate complex in the nucleus involving multiple proteins, and PIP_n_s thus directly affect cancer cell motility. The distinct nuclear PIP_n_-p53 signaling pathway, independent of canonical membrane-bound AKT activation and unaffected by existing PI3K inhibitors, highlights the potential for innovative therapeutic interventions that target these specific interactions in cancer treatment.

## Conclusion

The PIP_n_-p53 signalosome has emerged as a crucial regulator of cancer cell motility by providing the missing link between the independent relationships of this cellular process with both p53 and PIP_n_ signaling, particularly in metastasis. This review has shown that nuclear PIP_n_s, such as PtdIns4P, PtdIns(4,5)P(, and PtdIns(3,4,5)P’, interact with both wild type and mutant p53 to form signaling complexes that regulate cytoskeletal dynamics, cell adhesion, and nuclear AKT activation (Fig. 5). These interactions are essential in controlling the migration and invasion of cancer cells, with p53 acting as a critical scaffold in the nucleus to stabilize signaling pathways. Disruption of this signalosome through p53 mutations or altered PIP_n_ signaling promotes increased cell motility and metastasis, making this axis a promising target for therapeutic interventions.

The involvement of nuclear PIP_n_s in both stabilizing p53 and activating AKT re”ects a non-transcriptional function of p53 that is triggered under stress conditions (Chen et al., 2020, 2022; Choi et al., 2019). This mechanism operates in both wild type and mutant p53 contexts but is often amplified in cancer cells harboring mutant p53 due to the co-expression of nuclear PIP_n_ pathway components. Importantly, recent studies suggest that p53 is required for efficient nuclear PIP_n_-mediated AKT activation under genotoxic stress, serving to organize the signaling complex and direct lipid channeling (Chen et al., 2022). However, the apparent contradiction between p53 stabilization and AKT activation can be reconciled by considering regulatory factors, including differential protein interactions, post-translational modifications, or the temporal dynamics of signalosome assembly, such as proximity to the PtdIns(3,4,5)P_3_ phosphatase PTEN or nuclear AKT phosphatases (Chen et al., 2022; Chibaya et al., 2021; Huang et al., 2012; Ogawara et al., 2002; Zhang et al., 2011). These modulators likely dictate whether the net outcome favors tumor suppression or survival signaling.

To further elucidate the functional significance of this pathway, it is essential to consider the role of nuclear AKT activity in driving cancer cell motility. While AKT is traditionally associated with plasma membrane signaling, its nuclear functions have become increasingly recognized as key regulators of migration and invasion (Chen et al., 2022; Lee et al., 2008). Nuclear AKT phosphorylates transcription factors such as FOXO proteins, altering gene expression programs that in”uence cytoskeletal organization and cell adhesion (Hou et al., 2025b; Zhang et al., 2011). These transcriptional changes contribute to enhanced motility, particularly in meta-static cancer cells. Notably, aberrant nuclear AKT activation within the PIP_n_-p53 signalosome has been linked to increased invasiveness, further underscoring its role as a central mediator of cell migration (Chen et al., 2022; Hou et al., 2025b). Understanding the interplay between nuclear PIP_n_s, p53, and AKT in this context provides new insights into metastasis and highlights potential therapeutic strategies targeting this axis.

Combining PI3K/AKT inhibitors with agents that restore p53 function could produce a dual inhibitory effect on cancer cell migration (Abraham and O’Neill, 2014; Singh et al., 2002; Song et al., 2015; Turner et al., 2013). Cancer metastasis could be more effectively mitigated by blocking both the upstream activation of motility- related pathways (via PI3K inhibition) and restoring the ability of p53 to suppress cell motility. This combined approach may also enhance apoptosis in cancer cells, as p53 reactivation would regain its role in promoting cell death, while PI3K inhibition would reduce survival signaling. While most PI3K inhibitors, such as pan-PI3K inhibitors (e.g., BKM120 (buparlisib), GDC-0941), broadly suppress PI3K activity, recent studies have identified compounds with preferential nuclear activity (Sarker et al., 2015). For example, PI3Kα-specific inhibitors like BYL719 (alpelisib) have been shown to affect nuclear PtdIns(3,4,5)P’ signaling, thereby modulating nuclear AKT activation in colorectal cancer (Palmieri et al., 2023). However, these effects may be cancer type-specific. In breast cancer cells, for instance, the nuclear PI3K isoform IPMK is responsible for generating nuclear PtdIns(3,4,5)P’ and activating AKT (Chen et al., 2022). In this context, neither the panPI3K inhibitor BKM120 (buparlisib) nor the PI3Kα-specific inhibitor BYL719 (alpelisib) is effective, underscoring the need to develop more versatile PI3K inhibitors that also target non-canonical isoforms such as IPMK (Chen et al., 2022).

Additionally, PI4KIIα is intricately linked to focal adhesion dynamics and plays a significant role in maintaining the stability of p53 (Carrillo et al., 2023; Sun et al., 2023). Targeting PI4KIIα to inhibit focal adhesion formation could be paired with therapies to reactivate wild-type p53 (Bura et al., 2023; Carrillo et al., 2023; Gozzelino et al., 2020). This combination strategy decreases the cancer cells’ ability to establish stable attachments to the extra-cellular matrix, which is essential for migration and invasion, and it also suppresses pro-metastatic signals arising from mutant p53. By disrupting both the structural components of cell movement and the regulatory pathways in”uenced by p53, this integrated therapeutic approach holds promise for effectively reducing metastatic potential and improving treatment outcomes for patients with aggressive cancers. Further investigation into the structural dynamics of the PIP_n_-p53 signalosome is needed to understand how these complexes regulate nuclear processes comprehensively. Detailed structural studies could reveal new therapeutic targets within this network.

Since nuclear PIP_n_ signaling is less understood than cytoplasmic signaling, more research is needed to develop specific inhibitors that target nuclear PIP_n_s without affecting essential cytoplasmic functions. Future research will delve into the molecular mechanisms of these signalosomes, their role as biomarkers, and the development of targeted therapies.

A deeper understanding of how nuclear PIP_n_s interact with p53 and other nuclear proteins is essential for unraveling the complex regulation of cancer cell motility. Studies should explore the structural and functional details of the nuclear PIP_n_-p53 signalosome, particularly its role in mediating the nuclear localization and activity of essential signaling proteins.

## References

[pwaf043-B1] Abraham AG , O’Neill E. PI3K/Akt-mediated regulation of p53 in cancer. Biochem Soc Trans 2014;42:798–803. 25109960 10.1042/BST20140070

[pwaf043-B2] Agarwal ML , Taylor WR, Chernov MV et al The p53 network. J Biol Chem 1998;273:1–4. 9417035 10.1074/jbc.273.1.1

[pwaf043-B3] Ahmed NN , Franke TF, Bellacosa A et al The proteins encoded by c-akt and v-akt differ in post-translational modification, subcellular localization and oncogenic potential. Oncogene 1993;8:1957–1963. 8510938

[pwaf043-B4] Alli-Balogun GO , Gewinner CA, Jacobs R et al Phosphatidylinositol 4-kinase IIβ negatively regulates invadopodia formation and suppresses an invasive cellular phenotype. Mol Biol Cell 2016;27:4033–4042. 27798239 10.1091/mbc.E16-08-0564 PMC5156544

[pwaf043-B5] Araki K , Ebata T, Guo AK et al p53 regulates cytoskeleton remodeling to suppress tumor progression. Cell Mol Life Sci 2015;72:4077–4094. 26206378 10.1007/s00018-015-1989-9 PMC11114009

[pwaf043-B6] Ashlin TG , Blunsom NJ, Cockcroft S. Courier service for phosphatidylinositol: PITPs deliver on demand. Biochim Biophys Acta Mol Cell Biol Lipids 2021;1866:158985. 34111527 10.1016/j.bbalip.2021.158985 PMC8266687

[pwaf043-B7] Balla T. Phosphoinositides: tiny lipids with giant impact on cell regulation. Physiol Rev 2013;93:1019–1137. 23899561 10.1152/physrev.00028.2012 PMC3962547

[pwaf043-B8] Balla A , Balla T. Phosphatidylinositol 4-kinases: old enzymes with emerging functions. Trends Cell Biol 2006;16:351–361. 16793271 10.1016/j.tcb.2006.05.003

[pwaf043-B9] Banfic H , Visnjic D, Mise N et al Epidermal growth factor stimulates translocation of the class II phosphoinositide 3-kinase PI3K-C2β to the nucleus. Biochem J 2009;422:53–60. 19496756 10.1042/BJ20090654

[pwaf043-B10] Barlow CA , Laishram RS, Anderson RA. Nuclear phosphoinositides: a signaling enigma wrapped in a compartmental conundrum. Trends Cell Biol 2010;20:25–35. 19846310 10.1016/j.tcb.2009.09.009 PMC2818233

[pwaf043-B11] Blind RD , Suzawa M, Ingraham HA. Direct modification and activation of a nuclear receptor-PIP_2_ complex by the inositol lipid kinase IPMK. Sci Signal 2012;5:ra44. 22715467 10.1126/scisignal.2003111 PMC3395721

[pwaf043-B12] Blunsom NJ , Cockcroft S. CDP-diacylglycerol synthases (CDS): gateway to phosphatidylinositol and cardiolipin synthesis. Front Cell Dev Biol 2020a;8:63. 32117988 10.3389/fcell.2020.00063 PMC7018664

[pwaf043-B13] Blunsom NJ , Cockcroft S. Phosphatidylinositol synthesis at the endoplasmic reticulum. Biochim Biophys Acta Mol Cell Biol Lipids 2020b;1865:158471. 31173893 10.1016/j.bbalip.2019.05.015

[pwaf043-B14] Boronenkov IV , Loijens JC, Umeda M et al Phosphoinositide signaling pathways in nuclei are associated with nuclear speckles containing pre-mRNA processing factors. Mol Biol Cell 1998;9:3547–3560. 9843587 10.1091/mbc.9.12.3547 PMC25675

[pwaf043-B15] Bunce MW , Boronenkov IV, Anderson RA. Coordinated activation of the nuclear ubiquitin ligase Cul3-SPOP by the generation of phosphatidylinositol 5-phosphate. J Biol Chem 2008;283:8678–8686. 18218622 10.1074/jbc.M710222200

[pwaf043-B16] Bura A , Čabrijan S, Đurić I et al A plethora of functions condensed into tiny phospholipids: the story of PI4P and PI(4,5)P_2_. Cells 2023;12:1411. 37408244 10.3390/cells12101411 PMC10216963

[pwaf043-B17] Butera A , Amelio I. Deciphering the significance of p53 mutant proteins. Trends Cell Biol 2025;35:258–268. 38960851 10.1016/j.tcb.2024.06.003

[pwaf043-B18] Carrillo ND , Chen M, Wen T et al Lipid transfer proteins and a PI 4-kinase initiate nuclear phosphoinositide signaling. BioRxiv 2025:2023.05.08.539894. 10.1016/j.jbc.2026.113123 PMC13260207 42107639

[pwaf043-B19] Chakrabarti R , Sanyal S, Ghosh A et al Phosphatidylinositol-4-phosphate 5-kinase 1α modulates ribosomal RNA gene silencing through its interaction with histone H3 Lysine 9 trimethylation and heterochromatin protein HP1-α. J Biol Chem 2015;290:20893–20903. 26157143 10.1074/jbc.M114.633727 PMC4543650

[pwaf043-B20] Chang CJ , Chao CH, Xia W et al p53 regulates epithelial- mesenchymal transition and stem cell properties through modulating miRNAs. Nat Cell Biol 2011;13:317–323. 21336307 10.1038/ncb2173 PMC3075845

[pwaf043-B21] Charest PG , Firtel RA. Big roles for small GTPases in the control of directed cell movement. Biochem J 2007;401:377–390. 17173542 10.1042/BJ20061432 PMC1820805

[pwaf043-B22] Chen M , Choi S, Jung O et al The specificity of EGF-stimulated IQGAP1 scaffold towards the PI3K-Akt pathway is defined by the IQ3 motif. Sci Rep 2019;9:9126. 31235839 10.1038/s41598-019-45671-5 PMC6591252

[pwaf043-B23] Chen M , Wen T, Horn HT et al The nuclear phosphoinositide response to stress. Cell Cycle 2020;19:268–289. 31902273 10.1080/15384101.2019.1711316 PMC7028212

[pwaf043-B24] Chen M , Horn HT, Wen T et al Assessing *in situ* phosphoinositide- protein interactions through “uorescence proximity ligation assay in cultured cells. Methods Mol Biol 2021;2251:133–142. 33481236 10.1007/978-1-0716-1142-5_9 PMC9789737

[pwaf043-B25] Chen M , Choi S, Wen T et al A p53–phosphoinositide signalosome regulates nuclear AKT activation. Nat Cell Biol 2022;24:1099–1113. 35798843 10.1038/s41556-022-00949-1 PMC9833102

[pwaf043-B26] Chen C , Chen M, Wen T et al Regulation of NRF2 by Phosphoinositides and small heat shock proteins. bioRxiv 2024:2023.10.26.564194. 10.1016/j.jbc.2025.110367 PMC12273567 40516875

[pwaf043-B27] Cheng GZ , Zhang W, Wang L-H. Regulation of cancer cell survival, migration, and invasion by Twist: AKT2 comes to interplay. Cancer Res 2008;68:957–960. 18281467 10.1158/0008-5472.CAN-07-5067

[pwaf043-B28] Chibaya L , Karim B, Zhang H et al Mdm2 phosphorylation by Akt regulates the p53 response to oxidative stress to promote cell proliferation and tumorigenesis. Proc Natl Acad Sci USA 2021;118:e2003193118. 33468664 10.1073/pnas.2003193118 PMC7848548

[pwaf043-B29] Choi BH , Chen Y, Dai W. Chromatin PTEN is involved in DNA damage response partly through regulating Rad52 sumoylation. Cell Cycle 2013;12:3442–3447. 24047694 10.4161/cc.26465 PMC3895432

[pwaf043-B30] Choi S , Hedman AC, Sayedyahossein S et al Agonist-stimulated phosphatidylinositol-3,4,5-trisphosphate generation by scaffolded phosphoinositide kinases. Nat Cell Biol 2016;18:1324–1335. 27870828 10.1038/ncb3441 PMC5679705

[pwaf043-B31] Choi S , Chen M, Cryns VL et al A nuclear phosphoinositide kinase complex regulates p53. Nat Cell Biol 2019;21:462–475. 30886346 10.1038/s41556-019-0297-2 PMC7017954

[pwaf043-B32] Cingolani LA , Goda Y. Actin in action: the interplay between the actin cytoskeleton and synaptic efficacy. Nat Rev Neurosci 2008;9:344–356. 18425089 10.1038/nrn2373

[pwaf043-B33] Cocco L , Gilmour RS, Ognibene A et al Synthesis of polyphosphoinositides in nuclei of Friend cells. Evidence for polyphosphoinositide metabolism inside the nucleus which changes with cell differentiation. Biochem J 1987;248:765–770. 2829840 10.1042/bj2480765 PMC1148615

[pwaf043-B34] Cocco L , Rubbini S, Manzoli L et al Inositides in the nucleus: presence and characterisation of the isozymes of phospholipase “ family in NIH 3T3 cells. Biochim Biophys Acta 1999;1438:295–299. 10320812 10.1016/s1388-1981(99)00061-x

[pwaf043-B35] Coutts AS , Weston L, La Thangue NB. A transcription co- factor integrates cell adhesion and motility with the p53 response. Proc Natl Acad Sci USA 2009;106:19872–19877. 19897726 10.1073/pnas.0906785106 PMC2785259

[pwaf043-B36] de Graaf P , Klapisz EE, Schulz TK et al Nuclear localization of phosphatidylinositol 4-kinase “. J Cell Sci 2002;115:1769–1775. 11950893 10.1242/jcs.115.8.1769

[pwaf043-B37] Deleris P , Bacqueville D, Gayral S et al SHIP-2 and PTEN are expressed and active in vascular smooth muscle cell nuclei, but only SHIP-2 is associated with nuclear speckles. J Biol Chem 2003;278:38884–38891. 12847108 10.1074/jbc.M300816200

[pwaf043-B38] de Vries A , Flores ER, Miranda B et al Targeted point mutations of *p53* lead to dominant-negative inhibition of wild-type p53 function. Proc Natl Acad Sci USA 2002;99:2948–2953. 11867759 10.1073/pnas.052713099 PMC122453

[pwaf043-B39] Didichenko SA , Thelen M. Phosphatidylinositol 3-kinase c2α contains a nuclear localization sequence and associates with nuclear speckles. J Biol Chem 2001;276:48135–48142. 11606566 10.1074/jbc.M104610200

[pwaf043-B40] Ehm P , Nalaskowski MM, Wundenberg T et al The tumor suppressor SHIP1 colocalizes in nucleolar cavities with p53 and components of PML nuclear bodies. Nucleus 2015;6:154–164. 25723258 10.1080/19491034.2015.1022701 PMC4615814

[pwaf043-B41] Elis W , Triantafellow E, Wolters NM et al Down-regulation of class II phosphoinositide 3-kinase alpha expression below a critical threshold induces apoptotic cell death. Mol Cancer Res 2008;6:614–623. 18403640 10.1158/1541-7786.MCR-07-0262

[pwaf043-B42] Elong Edimo W , Derua R, Janssens V et al Evidence of SHIP2 Ser132 phosphorylation, its nuclear localization and stability. Biochem J 2011;439:391–401. 21770892 10.1042/BJ20110173

[pwaf043-B43] Enomoto A , Murakami H, Asai N et al Akt/PKB regulates actin organization and cell motility via Girdin/APE. Dev Cell 2005;9:389–402. 16139227 10.1016/j.devcel.2005.08.001

[pwaf043-B44] Faenza I , Fiume R, Piazzi M et al Nuclear inositide specific phospholipase C signalling – interactions and activity. FEBS J 2013;280:6311–6321. 23890371 10.1111/febs.12450

[pwaf043-B45] Gadéa G , Lapasset L, Gauthier-Rouvière C et al Regulation of Cdc42-mediated morphological effects: a novel function for p53. EMBO J 2002;21:2373–2382. 12006490 10.1093/emboj/21.10.2373 PMC126005

[pwaf043-B46] Gadea G , de Toledo M, Anguille C et al Loss of p53 promotes RhoA–ROCK-dependent cell migration and invasion in 3D matrices. J Cell Biol 2007;178:23–30. 17606864 10.1083/jcb.200701120 PMC2064414

[pwaf043-B47] Gonzalez E , McGraw TE. The Akt kinases: isoform specificity in metabolism and cancer. Cell cycle 2009;8:2502–2508. 19597332 10.4161/cc.8.16.9335 PMC2997486

[pwaf043-B48] Govindarajan B , Sbrissa D, Pressprich M et al Adaptor proteins mediate CXCR4 and PI4KA crosstalk in prostate cancer cells and the significance of PI4KA in bone tumor growth. Sci Rep 2023;13:20634. 37996444 10.1038/s41598-023-47633-4 PMC10667255

[pwaf043-B49] Gozani O , Karuman P, Jones DR et al The PHD Finger of the chromatin-associated protein ING2 functions as a nuclear phosphoinositide receptor. Cell 2003;114:99–111. 12859901 10.1016/s0092-8674(03)00480-x

[pwaf043-B50] Gozzelino L , De Santis MC, Gulluni F et al PI(3,4)P2 signaling in cancer and metabolism. Front Oncol 2020;10:360. 32296634 10.3389/fonc.2020.00360 PMC7136497

[pwaf043-B51] Guo F , Gao Y, Wang L et al p19Arf-p53 tumor suppressor pathway regulates cell motility by suppression of phosphoinositide 3-kinase and Rac1 GTPase activities. J Biol Chem 2003;278:14414–14419. 12578823 10.1074/jbc.M300341200

[pwaf043-B52] Hao Y , He B, Wu L et al Nuclear translocation of p85β promotes tumorigenesis of PIK3CA helical domain mutant cancer. Nat Commun 2022;13:1974. 35418124 10.1038/s41467-022-29585-x PMC9007954

[pwaf043-B53] Hassin O , Oren M. Drugging p53 in cancer: one protein, many targets. Nat Rev Drug Discovery 2023;22:127–144. 36216888 10.1038/s41573-022-00571-8 PMC9549847

[pwaf043-B54] Hollstein M , Sidransky D, Vogelstein B et al p53 mutations in human cancers. Science 1991;253:49–53. 1905840 10.1126/science.1905840

[pwaf043-B55] Hou X , Chen Y, Carrillo ND et al Phosphoinositide signaling at the cytoskeleton in the regulation of cell dynamics. Cell Death Dis 2025a;16:296. 40229242 10.1038/s41419-025-07616-x PMC11997203

[pwaf043-B56] Hou X , Ren C, Jin J et al Phosphoinositide signalling in cell motility and adhesion. Nat Cell Biol 2025b;27:736–748. 40169755 10.1038/s41556-025-01647-4

[pwaf043-B57] Hsuan J , Cockcroft S. The PITP family of phosphatidylinositol transfer proteins. Genome Biol 2001;2:REVIEWS3011. 11574064 10.1186/gb-2001-2-9-reviews3011 PMC138965

[pwaf043-B58] Huang Y , Jeong JS, Okamura J et al Global tumor protein p53/ p63 interactome: making a case for cisplatin chemoresistance. Cell Cycle 2012;11:2367–2379. 22672905 10.4161/cc.20863 PMC3383596

[pwaf043-B59] Isaji T , Im S, Kameyama A et al A complex between phosphatidylinositol 4-kinase IIα and integrin α3β1 is required for N-glycan sialylation in cancer cells. J Biol Chem 2019;294:4425–4436. 30659093 10.1074/jbc.RA118.005208 PMC6433072

[pwaf043-B60] Jean S , Kiger AA. Classes of phosphoinositide 3-kinases at a glance. J Cell Sci 2014;127:923–928. 24587488 10.1242/jcs.093773 PMC3937771

[pwaf043-B61] Jung O , Baek M-j, Wooldrik C et al Nuclear phosphoinositide signaling promotes YAP/TAZ-TEAD transcriptional activity in breast cancer. EMBO J 2024;43:1740–1769. 38565949 10.1038/s44318-024-00085-6 PMC11066040

[pwaf043-B62] Kakuk A , Friedlander E, Vereb G et al Nucleolar localization of phosphatidylinositol 4-kinase PI4K230 in various mammalian cells. Cytometry A 2006;69:1174–1183. 17131383 10.1002/cyto.a.20347

[pwaf043-B63] Kakuk A , Friedlander E, Vereb G et al Nuclear and nucleolar localization signals and their targeting function in phosphatidylinositol 4-kinase PI4K230. Exp Cell Res 2008;314:2376–2388. 18585705 10.1016/j.yexcr.2008.05.006

[pwaf043-B64] Kandoth C , McLellan MD, Vandin F et al Mutational landscape and significance across 12 major cancer types. Nature 2013;502:333–339. 24132290 10.1038/nature12634 PMC3927368

[pwaf043-B65] Karlsson T , Altankhuyag A, Dobrovolska O et al A polybasic motif in ErbB3-binding protein 1 (EBP1) has key functions in nucleolar localization and polyphosphoinositide interaction. Biochem J 2016;473:2033–2047. 27118868 10.1042/BCJ20160274 PMC4941749

[pwaf043-B66] Ke M , Zhu H, Lin Y et al Actin-related protein 2/3 complex subunit 1B promotes ovarian cancer progression by regulating the AKT/PI3K/mTOR signaling pathway. J Transl Int Med 2024;12:406–423. 39360160 10.2478/jtim-2024-0025 PMC11444474

[pwaf043-B67] Kennedy MC , Lowe SW. Mutant p53: it’s not all one and the same. Cell Death Differ 2022;29:983–987. 35361963 10.1038/s41418-022-00989-y PMC9090915

[pwaf043-B68] Kouchi Z , Fujiwara Y, Yamaguchi H et al Phosphatidylinositol 5-phosphate 4-kinase type II beta is required for vitamin D receptor-dependent E-cadherin expression in SW480 cells. Biochem Biophys Res Commun 2011;408:523–529. 21514270 10.1016/j.bbrc.2011.04.045

[pwaf043-B69] Kruse J-P, Gu W. Modes of p53 Regulation. Cell 2009; 137:609–622. 19450511 10.1016/j.cell.2009.04.050 PMC3737742

[pwaf043-B70] Kubbutat MH , Jones SN, Vousden KH. Regulation of p53 stability by Mdm2. Nature 1997;387:299–303. 9153396 10.1038/387299a0

[pwaf043-B71] Kumar A , Redondo-Munoz J, Perez-Garcia V et al Nuclear but not cytosolic phosphoinositide 3-kinase beta has an essential function in cell survival. Mol Cell Biol 2011;31:2122–2133. 21383062 10.1128/MCB.01313-10 PMC3133359

[pwaf043-B72] Kunrath-Lima M , de Miranda MC, Ferreira ADF et al Phospholipase C delta 4 (PLCδ4) is a nuclear protein involved in cell proliferation and senescence in mesenchymal stromal stem cells. Cell Signal 2018;49:59–67. 29859928 10.1016/j.cellsig.2018.05.011 PMC6095203

[pwaf043-B73] Langerød A , Zhao H, Borgan O et al TP53 mutation status and gene expression profiles are powerful prognostic markers of breast cancer. Breast Cancer Res 2007;9:R30. 17504517 10.1186/bcr1675 PMC1929092

[pwaf043-B74] Lattanzio R , Iezzi M, Sala G et al PLC-gamma-1 phosphorylation status is prognostic of metastatic risk in patients with early-stage Luminal-A and -B breast cancer sub-types. BMC Cancer 2019;19:747. 31362705 10.1186/s12885-019-5949-x PMC6668079

[pwaf043-B75] Lee SB , Xuan Nguyen TL, Choi JW et al Nuclear Akt interacts with B23/NPM and protects it from proteolytic cleavage, enhancing cell survival. Proc Natl Acad Sci USA 2008;105:16584–16589. 18931307 10.1073/pnas.0807668105 PMC2569968

[pwaf043-B76] Li Y , Luan C. PLCE1 promotes the invasion and migration of esophageal cancer cells by up-regulating the PKCα/NF-κB pathway. Yonsei Med J 2018;59:1159–1165. 30450849 10.3349/ymj.2018.59.10.1159 PMC6240569

[pwaf043-B77] Li G , Nelsen C, Hendrickson EA. Ku86 is essential in human somatic cells. Proc Natl Acad Sci USA 2002;99:832–837. 11792868 10.1073/pnas.022649699 PMC117391

[pwaf043-B78] Li P , Wang D, Li H et al Identification of nucleolus-localized PTEN and its function in regulating ribosome biogenesis. Mol Biol Rep 2014;41:6383–6390. 24969487 10.1007/s11033-014-3518-6

[pwaf043-B79] Li J , Gao Z, Zhao D et al PI-273, a substrate- competitive, specific small-molecule inhibitor of PI4KIIα, inhibits the growth of breast cancer cells. Cancer Res 2017;77:6253–6266. 28827373 10.1158/0008-5472.CAN-17-0484

[pwaf043-B80] Manning BD , Toker A. AKT/PKB signaling: navigating the network. Cell 2017;169:381–405. 28431241 10.1016/j.cell.2017.04.001 PMC5546324

[pwaf043-B81] Mantovani F , Collavin L, Del Sal G. Mutant p53 as a guardian of the cancer cell. Cell Death Differ 2019;26:199–212. 30538286 10.1038/s41418-018-0246-9 PMC6329812

[pwaf043-B82] Manzoli FA , Maraldi NM, Cocco L et al Chromatin phospholipids in normal and chronic lymphocytic leukemia lymphocytes. Cancer Res 1977;37:843–849. 300041

[pwaf043-B83] Marques M , Kumar A, Poveda AM et al Specific function of phosphoinositide 3-kinase beta in the control of DNA replication. Proc Natl Acad Sci USA 2009;106:7525–7530. 19416922 10.1073/pnas.0812000106 PMC2678616

[pwaf043-B84] Martelli AM , Tabellini G, Bressanin D et al The emerging multiple roles of nuclear Akt. Biochim Biophys Acta 2012;1823:2168–2178. 22960641 10.1016/j.bbamcr.2012.08.017

[pwaf043-B85] May P , May E. Twenty years of p53 research: structural and functional aspects of the p53 protein. Oncogene 1999;18:7621–7636. 10618702 10.1038/sj.onc.1203285

[pwaf043-B86] Mazloumi Gavgani F , Smith Arnesen V, Jacobsen RG et al Class I Phosphoinositide 3-Kinase PIK3CA/p110β and PIK3CB/p110β Isoforms in Endometrial Cancer. Int J Mol Sci 2018;19:3931. 30544563 10.3390/ijms19123931 PMC6321576

[pwaf043-B87] Meier R , Alessi DR, Cron P et al Mitogenic activation, phosphorylation, and nuclear translocation of protein kinase Bbeta. J Biol Chem 1997;272:30491–30497. 9374542 10.1074/jbc.272.48.30491

[pwaf043-B88] Mellman DL , Gonzales ML, Song C et al A PtdIns4, 5P2-regulated nuclear poly (A) polymerase controls expression of select mRNAs. Nature 2008;451:1013–1017. 18288197 10.1038/nature06666

[pwaf043-B89] Merino-Casallo F , Gomez-Benito MJ, Hervas-Raluy S et al Unravelling cell migration: defining movement from the cell surface. Cell Adh Migr 2022;16:25–64. 35499121 10.1080/19336918.2022.2055520 PMC9067518

[pwaf043-B90] Meza-Sosa KF , Miao R, Navarro F et al SPARCLE, a p53-induced lncRNA, controls apoptosis after genotoxic stress by promoting PARP-1 cleavage. Mol Cell 2022;82:785–802.e10. 35104452 10.1016/j.molcel.2022.01.001 PMC10392910

[pwaf043-B91] Mizuarai S , Yamanaka K, Kotani H. Mutant p53 induces the GEF-H1 oncogene, a guanine nucleotide exchange factor- H1 for RhoA, resulting in accelerated cell proliferation in tumor cells. Cancer Res 2006;66:6319–6326. 16778209 10.1158/0008-5472.CAN-05-4629

[pwaf043-B92] Morrow AA , Alipour MA, Bridges D et al The lipid kinase PI4KIIIβ is highly expressed in breast tumors and activates Akt in cooperation with Rab11a. Mol Cancer Res 2014;12:1492–1508. 24962317 10.1158/1541-7786.MCR-13-0604

[pwaf043-B93] Muller PA , Vousden KH, Norman JC. p53 and its mutants in tumor cell migration and invasion. J Cell Biol 2011;192:209–218. 21263025 10.1083/jcb.201009059 PMC3172183

[pwaf043-B94] Neri LM , Bortul R, Borgatti P et al Proliferating or differentiating stimuli act on different lipid-dependent signaling pathways in nuclei of human leukemia cells. Mol Biol Cell 2002;13:947–964. 11907274 10.1091/mbc.01-02-0086 PMC99611

[pwaf043-B95] Ogawara Y , Kishishita S, Obata T et al Akt enhances Mdm2-mediated ubiquitination and degradation of p53. J Biol Chem 2002;277:21843–21850. 11923280 10.1074/jbc.M109745200

[pwaf043-B96] Okazaki R. Role of p53 in regulating radiation responses. Life (Basel, Switzerland) 2022;12:1099. 35888186 10.3390/life12071099 PMC9319710

[pwaf043-B97] Olivier M , Hollstein M, Hainaut P. TP53 mutations in human cancers: origins, consequences, and clinical use. Cold Spring Harb Perspect Biol 2010;2:a001008. 20182602 10.1101/cshperspect.a001008 PMC2827900

[pwaf043-B98] Oren M , Prives C. p53: a tale of complexity and context. Cell 2024;187:1569–1573. 38552605 10.1016/j.cell.2024.02.043

[pwaf043-B99] Owusu Obeng E , Rusciano I, Marvi MV et al Phosphoinositide-dependent signaling in cancer: a focus on phospholipase C isozymes. Int J Mol Sci 2020;21:2581. 32276377 10.3390/ijms21072581 PMC7177890

[pwaf043-B100] Palmieri M , Catimel B, Mouradov D et al PI3Kα translocation mediates nuclear PtdIns(3,4,5)P_3_ effector signaling in colorectal cancer. Mol Cell Proteomics 2023;22:100529. 36931626 10.1016/j.mcpro.2023.100529 PMC10130476

[pwaf043-B101] Pedicone C , Meyer ST, Chisholm JD et al Targeting SHIP1 and SHIP2 in cancer. Cancers (Basel) 2021;13:890. 33672717 10.3390/cancers13040890 PMC7924360

[pwaf043-B102] Posor Y , Jang W, Haucke V. Phosphoinositides as membrane organizers. Nat Rev Mol Cell Biol 2022;23:797–816. 35589852 10.1038/s41580-022-00490-x PMC9117997

[pwaf043-B103] Razmara M , Heldin CH, Lennartsson J. Platelet-derived growth factor-induced Akt phosphorylation requires mTOR/Rictor and phospholipase C-$1, whereas S6 phosphorylation depends on mTOR/Raptor and phospholipase D. Cell Commun Signal 2013;11:3. 23311350 10.1186/1478-811X-11-3 PMC3560233

[pwaf043-B104] Ren C , Carrillo ND, Cryns VL et al Environmental pollutants and phosphoinositide signaling in autoimmunity. J Hazard Mater 2024;465:133080. 38091799 10.1016/j.jhazmat.2023.133080 PMC10923067

[pwaf043-B105] Rose HG , Frenster JH. Composition and metabolism of lipids within repressed and active chromatin of interphase lymphocytes. Biochim Biophys Acta 1965;106:577–591. 5881334 10.1016/0005-2760(65)90073-1

[pwaf043-B106] Russo A , Okur MN, Bosland M et al Phosphatidylinositol 3-kinase, class 2 beta (PI3KC2β) isoform contributes to neuroblastoma tumorigenesis. Cancer Lett 2015;359:262–268. 25622909 10.1016/j.canlet.2015.01.026 PMC4351744

[pwaf043-B107] Saji M , Vasko V, Kada F et al Akt1 contains a functional leucine- rich nuclear export sequence. Biochem Biophys Res Commun 2005;332:167–173. 15896313 10.1016/j.bbrc.2005.04.109

[pwaf043-B108] Sale E , Sale G. Protein kinase B: signalling roles and therapeutic targeting. Cell Mol Life Sci 2008;65:113–127. 17952368 10.1007/s00018-007-7274-9 PMC11131913

[pwaf043-B109] Sarker D , Ang JE, Baird R et al First-in-human phase I study of pictilisib (GDC-0941), a potent pan-class I phosphatidylinositol-3-kinase (PI3K) inhibitor, in patients with advanced solid tumors. Clin Cancer Res 2015;21:77–86. 25370471 10.1158/1078-0432.CCR-14-0947 PMC4287394

[pwaf043-B110] Sbrissa D , Semaan L, Govindarajan B et al A novel cross-talk between CXCR4 and PI4KIIIα in prostate cancer cells. Oncogene 2019;38:332–344. 30111818 10.1038/s41388-018-0448-0 PMC6336684

[pwaf043-B111] Schill NJ , Anderson RA. Two novel phosphatidylinositol- 4-phosphate 5-kinase type Igamma splice variants expressed in human cells display distinctive cellular targeting. Biochem J 2009;422:473–482. 19548880 10.1042/BJ20090638 PMC2782315

[pwaf043-B112] Seok YM , Azam MA, Okamoto Y et al Enhanced Ca^2+-^dependent activation of phosphoinositide 3-kinase class IIα isoform-Rho axis in blood vessels of spontaneously hypertensive rats. Hypertension 2010;56:934–941. 20921425 10.1161/HYPERTENSIONAHA.110.160853

[pwaf043-B113] Shen WH , Balajee AS, Wang J et al Essential role for nuclear PTEN in maintaining chromosomal integrity. Cell 2007;128:157–170. 17218262 10.1016/j.cell.2006.11.042

[pwaf043-B114] Sigalotti L , Covre A, Fratta E et al Epigenetics of human cutaneous melanoma: setting the stage for new therapeutic strategies. J Transl Med 2010;8:1–22. 20540720 10.1186/1479-5876-8-56 PMC2901206

[pwaf043-B115] Sindić A , Aleksandrova A, Fields AP et al Presence and activation of nuclear phosphoinositide 3-kinase C2β during compensatory liver growth. J Biol Chem 2001;276:17754–17761. 11278304 10.1074/jbc.M006533200

[pwaf043-B116] Singh B , Reddy PG, Goberdhan A et al p53 regulates cell survival by inhibiting PIK3CA in squamous cell carcinomas. Genes Dev 2002;16:984–993. 11959846 10.1101/gad.973602 PMC152354

[pwaf043-B117] Sinkala M. Mutational landscape of cancer-driver genes across human cancers. Sci Rep 2023;13:12742. 37550388 10.1038/s41598-023-39608-2 PMC10406856

[pwaf043-B118] Smith CD , Wells W. Phosphorylation of rat liver nuclear envelopes. II. Characterization of *in vitro* lipid phosphorylation. J Biol Chem 1983;258:9368–9373. 6308005

[pwaf043-B119] Sone Y , Ito M, Shirakawa H et al Nuclear translocation of phospholipase C-zeta, an egg-activating factor, during early embryonic development. Biochem Biophys Res Commun 2005;330:690–694. 15809052 10.1016/j.bbrc.2005.03.032

[pwaf043-B120] Song R , Tian K, Wang W et al P53 suppresses cell proliferation, metastasis, and angiogenesis of osteosarcoma through inhibition of the PI3K/AKT/mTOR pathway. International Journal of Surgery (London, England) 2015;20:80–87. 25936826 10.1016/j.ijsu.2015.04.050

[pwaf043-B121] Srinivasan S , Wang F, Glavas S et al Rac and Cdc42 play distinct roles in regulating PI(3,4,5)P3 and polarity during neutrophil chemotaxis. J Cell Biol 2003;160:375–385. 12551955 10.1083/jcb.200208179 PMC2172671

[pwaf043-B122] Stallings JD , Tall EG, Pentyala S et al Nuclear translocation of phospholipase C-δ_1_ is linked to the cell cycle and nuclear phosphatidylinositol 4,5-bisphosphate. J Biol Chem 2005;280:22060–22069. 15809301 10.1074/jbc.M413813200

[pwaf043-B123] Stijf-Bultsma Y , Sommer L, Tauber M et al The basal transcription complex component TAF3 transduces changes in nuclear phosphoinositides into transcriptional output. Mol Cell 2015;58:453–467. 25866244 10.1016/j.molcel.2015.03.009 PMC4429956

[pwaf043-B124] Stuelten CH , Parent CA, Montell DJ. Cell motility in cancer invasion and metastasis: insights from simple model organisms. Nat Rev Cancer 2018;18:296–312. 29546880 10.1038/nrc.2018.15 PMC6790333

[pwaf043-B125] Sun Y , Turbin DA, Ling K et al Type I gamma phosphatidylinositol phosphate kinase modulates invasion and proliferation and its expression correlates with poor prognosis in breast cancer. Breast Cancer Res 2010;12:R6. 20074374 10.1186/bcr2471 PMC2880426

[pwaf043-B126] Sun Y , Isaji T, Oyama Y et al Focal-adhesion kinase regulates the sialylation of N-glycans via the PI4KIIα-PI4P pathway. J Biol Chem 2023;299:105051. 37451482 10.1016/j.jbc.2023.105051 PMC10406863

[pwaf043-B127] Szivak I , Lamb N, Heilmeyer LM. Subcellular localization and structural function of endogenous phosphorylated phosphatidylinositol 4-kinase (PI4K92). J Biol Chem 2006;281:16740–16749. 16606619 10.1074/jbc.M511645200

[pwaf043-B128] Sztacho M , Sobol M, Balaban C et al Nuclear phosphoinositides and phase separation: important players in nuclear compartmentalization. Advances in Biological Regulation 2019;71:111–117. 30249540 10.1016/j.jbior.2018.09.009

[pwaf043-B129] Tabellini G , Bortul R, Santi S et al Diacylglycerol kinase-theta is localized in the speckle domains of the nucleus. Exp Cell Res 2003;287:143–154. 12799190 10.1016/s0014-4827(03)00115-0

[pwaf043-B130] Tan X , Banerjee P, Pham EA et al PI4KIII” is a therapeutic target in chromosome 1q-amplified lung adenocarcinoma. Sci Transl Med 2020;12:eaax3772. 31969487 10.1126/scitranslmed.aax3772 PMC7702266

[pwaf043-B131] Tanaka K , Horiguchi K, Yoshida T et al Evidence that a phosphatidylinositol 3,4,5-trisphosphate-binding protein can function in nucleus. J Biol Chem 1999;274:3919–3922. 9933577 10.1074/jbc.274.7.3919

[pwaf043-B132] Thapa N , Chen M, Horn HT et al Phosphatidylinositol 3-kinase signalling is spatially organized at endosomal compartments by microtubule-associated protein 4. Nat Cell Biol 2020;22:1357–1370. 33139939 10.1038/s41556-020-00596-4 PMC8647654

[pwaf043-B133] Thapa N , Chen M, Cryns VL et al A p85 isoform switch enhances PI3K activation on endosomes by a MAP4- and PI3P-dependent mechanism. Cell Rep 2024;43:114119. 38630589 10.1016/j.celrep.2024.114119 PMC11380499

[pwaf043-B134] Toulany M , Iida M, Lettau K et al Targeting HER3-dependent activation of nuclear AKT improves radiotherapy of non-small cell lung cancer. Radiother Oncol 2022;174:92–100. 35839938 10.1016/j.radonc.2022.07.008 PMC10083767

[pwaf043-B135] Tran CS , Kersten J, Yan J et al Phosphatidylinositol 4-kinase III alpha governs cytoskeletal organization for invasiveness of liver cancer cells. Gastroenterology 2024;167:522–537. 38636680 10.1053/j.gastro.2024.04.009

[pwaf043-B136] Tribble EK , Ivanova PT, Grabon A et al Quantitative profiling of the endonuclear glycerophospholipidome of murine embryonic fibroblasts. J Lipid Res 2016;57:1492–1506. 27256690 10.1194/jlr.M068734 PMC4959864

[pwaf043-B137] Turner N , Moretti E, Siclari O et al Targeting triple negative breast cancer: Is p53 the answer? Cancer Treat Rev 2013;39:541–550. 23321033 10.1016/j.ctrv.2012.12.001

[pwaf043-B138] Vallejo-Díaz J , Chagoyen M, Olazabal-Morán M et al The opposing roles of PIK3R1/p85α and PIK3R2/p85” in cancer. Trends in Cancer 2019;5:233–244. 30961830 10.1016/j.trecan.2019.02.009

[pwaf043-B139] Vara JAF , Casado E, de Castro J et al PI3K/Akt signalling pathway and cancer. Cancer Treat Rev 2004;30:193–204. 15023437 10.1016/j.ctrv.2003.07.007

[pwaf043-B140] Vasko V , Saji M, Hardy E et al Akt activation and localisation correlate with tumour invasion and oncogene expression in thyroid cancer. J Med Genet 2004;41:161–170. 14985374 10.1136/jmg.2003.015339 PMC1735712

[pwaf043-B141] Visnjic D , Crljen V, Curic J et al The activation of nuclear phosphoinositide 3-kinase C2” in all-*trans*-retinoic acid- differentiated HL-60 cells. FEBS Lett 2002;529:268–274. 12372612 10.1016/s0014-5793(02)03357-4

[pwaf043-B142] Wainstein E , Maik-Rachline G, Blenis J et al AKTs do not translocate to the nucleus upon stimulation but AKT3 can constitutively signal from the nuclear envelope. Cell Rep 2022;41:111733. 36476861 10.1016/j.celrep.2022.111733

[pwaf043-B143] Walerych D , Napoli M, Collavin L et al The rebel angel: mutant p53 as the driving oncogene in breast cancer. Carcinogenesis 2012;33:2007–2017. 22822097 10.1093/carcin/bgs232 PMC3483014

[pwaf043-B144] Walerych D , Lisek K, Del Sal G. Mutant p53: one, no one, and one hundred thousand. Front Oncol 2015;5:289. 26734571 10.3389/fonc.2015.00289 PMC4685664

[pwaf043-B145] Wang Y-H, Sheetz MP. When PIP2 meets p53: nuclear phosphoinositide signaling in the DNA damage response. Front Cell Dev Biol 2022;10:903994. 35646908 10.3389/fcell.2022.903994 PMC9136457

[pwaf043-B146] Wang Y , Helland A, Holm R et al TP53 mutations in early- stage ovarian carcinoma, relation to long-term survival. Br J Cancer 2004a;90:678–685. 14760384 10.1038/sj.bjc.6601537 PMC2410156

[pwaf043-B147] Wang Y , Kringen P, Kristensen GB et al Effect of the codon 72 polymorphism (c.215G>C, p.Arg72Pro) in combination with somatic sequence variants in the TP53 gene on survival in patients with advanced ovarian carcinoma. Hum Mutat 2004b;24:21–34. 15221786 10.1002/humu.20055

[pwaf043-B148] Wen T , Chen M, Cryns VL et al Regulation of the poly(A) polymerase Star-PAP by a nuclear phosphoinositide signalosome. bioRxiv 2024:2024.07.01.601467.

[pwaf043-B149] Wong LH , Gatta AT, Levine TP. Lipid transfer proteins: the lipid commute via shuttles, bridges and tubes. Nat Rev Mol Cell Biol 2019;20:85–101. 30337668 10.1038/s41580-018-0071-5

[pwaf043-B150] Xu R , Sen N, Paul BD et al Inositol polyphosphate multikinase is a coactivator of p53-mediated transcription and cell death. Sci Signal 2013;6:ra22. 23550211 10.1126/scisignal.2003405 PMC4108196

[pwaf043-B151] Xue G , Hemmings BA. PKB/Akt–dependent regulation of cell motility. J Natl Cancer Inst 2013;105:393–404. 23355761 10.1093/jnci/djs648

[pwaf043-B152] Yang Y , Lee M, Fairn GD. Phospholipid subcellular localization and dynamics. J Biol Chem 2018;293:6230–6240. 29588369 10.1074/jbc.R117.000582 PMC5925819

[pwaf043-B153] Yeudall WA , Wrighton KH, Deb S. Mutant p53 in cell adhesion and motility. Methods Mol Biol 2013;962:135–146. 23150443 10.1007/978-1-62703-236-0_11

[pwaf043-B154] Yoeli-Lerner M , Yiu GK, Rabinovitz I et al Akt blocks breast cancer cell motility and invasion through the transcription factor NFAT. Mol Cell 2005;20:539–550. 16307918 10.1016/j.molcel.2005.10.033

[pwaf043-B155] Yoo SH , Huh YH, Huh SK et al Localization and projected role of phosphatidylinositol 4-kinases IIα and IIβ in inositol 1,4,5-trisphosphate-sensitive nucleoplasmic Ca^2+^ store vesicles. Nucleus 2014;5:341–351. 25482123 10.4161/nucl.29776 PMC4152348

[pwaf043-B156] Yue X , Zhang C, Zhao Y et al Gain-of-function mutant p53 activates small GTPase Rac1 through SUMOylation to promote tumor progression. Genes Dev 2017;31:1641–1654. 28947497 10.1101/gad.301564.117 PMC5647935

[pwaf043-B157] Zhang X , Tang N, Hadden TJ et al Akt, FoxO and regulation of apoptosis. Biochim Biophys Acta 2011;1813:1978–1986. 21440011 10.1016/j.bbamcr.2011.03.010

[pwaf043-B158] Zou J , Marjanovic J, Kisseleva MV et al Type I phosphatidylinositol- 4,5-bisphosphate 4-phosphatase regulates stress-induced apoptosis. Proc Natl Acad Sci USA 2007;104:16834–16839. 17940011 10.1073/pnas.0708189104 PMC2040409

